# The proposal of philosophical basis of the health care system

**DOI:** 10.1007/s11019-016-9717-2

**Published:** 2016-08-04

**Authors:** Andrzej Bielecki, Sylwia Nieszporska

**Affiliations:** 1 Wojtyła Institute – Scientific Foundation, Smoleńsk 29, 32-112 Kraków, Poland; 2 Faculty of Management, Częstochowa University of Technology, Armii Krajowej 19B, 42-200 Czestochowa, Poland

**Keywords:** Health care systems, Philosophical foundations, Personalism

## Abstract

The studies of health care systems are conducted intensively on various levels. They are important because the systems suffer from numerous pathologies. The health care is analyzed, first of all, in economic aspects but their functionality in the framework of systems theory is studied, as well. There are also attempts to work out some general values on which health care systems should be based. Nevertheless, the aforementioned studies, however, are fragmentary ones. In this paper holistic approach to the philosophical basis of health care is presented. The levels on which the problem can be considered are specified explicitly and relations between them are analyzed, as well. The philosophical basis on which the national health care systems could be based is proposed. Personalism is the basis for the proposal. First of all, the values, that are derived from the personalistic philosophy, are specified as the basic ones for health care systems. Then, general organizational and functional properties of the system are derived from the assumed values. The possibility of adaptation of solutions from other fields of social experiences are also mentioned. The existing health care systems are analyzed within the frame of the introduced proposal.

## Introduction

In most of the contemporary countries the health care system is organized on the national level which means that the state is responsible for its organization. All such types of health care systems, however, suffer from numerous pathologies which cause that they are more or less dysfunctional (Bar-Yam 2006; Bielecki and Nieszporska 2016; Bielecki and Stocki 2010; Krause 2013). Therefore, the systems are analyzed in various aspects, first of all, the economic ones. Their functionality are studied within the frames of systems theory (Bar-Yam 2006; Bielecki and Nieszporska 2016; Bielecki and Stocki 2010; Fahey et al. 2004; Homer and Hirsch 2006). In these papers the value of adopting a systems framework, in order to understand the complexities of the health care system, was demonstrated. There was proposed the concept mapping to identify key challenges to implementation of systems thinking and modeling in public health. The flows of information, care, and finance in the national health care system were studied by means of systemic analysis. There were also attempts to introduce the general values on which health care systems should be based. The aforementioned studies, however, are fragmentary ones. In this paper the philosophical basis on which the national health care systems, first of all in the European circle of culture,^1^ can be based, is discussed. The personalistic philosophy is proposed as the basis of the proposal which is put forward. It is obvious that the philosophical basis which concerns the health care system organization should be accordant with the basis which concern health as such. The concept of the basis for the health care system, presented in this paper, is agreeable with the idea that refers to the concept of health as such. According to the latter one, a successful definition of health needs, as the main point, an anthropological approach which, among others, takes into account the human being’s specific nature (Spijk 2015).

**Fig. 1:**
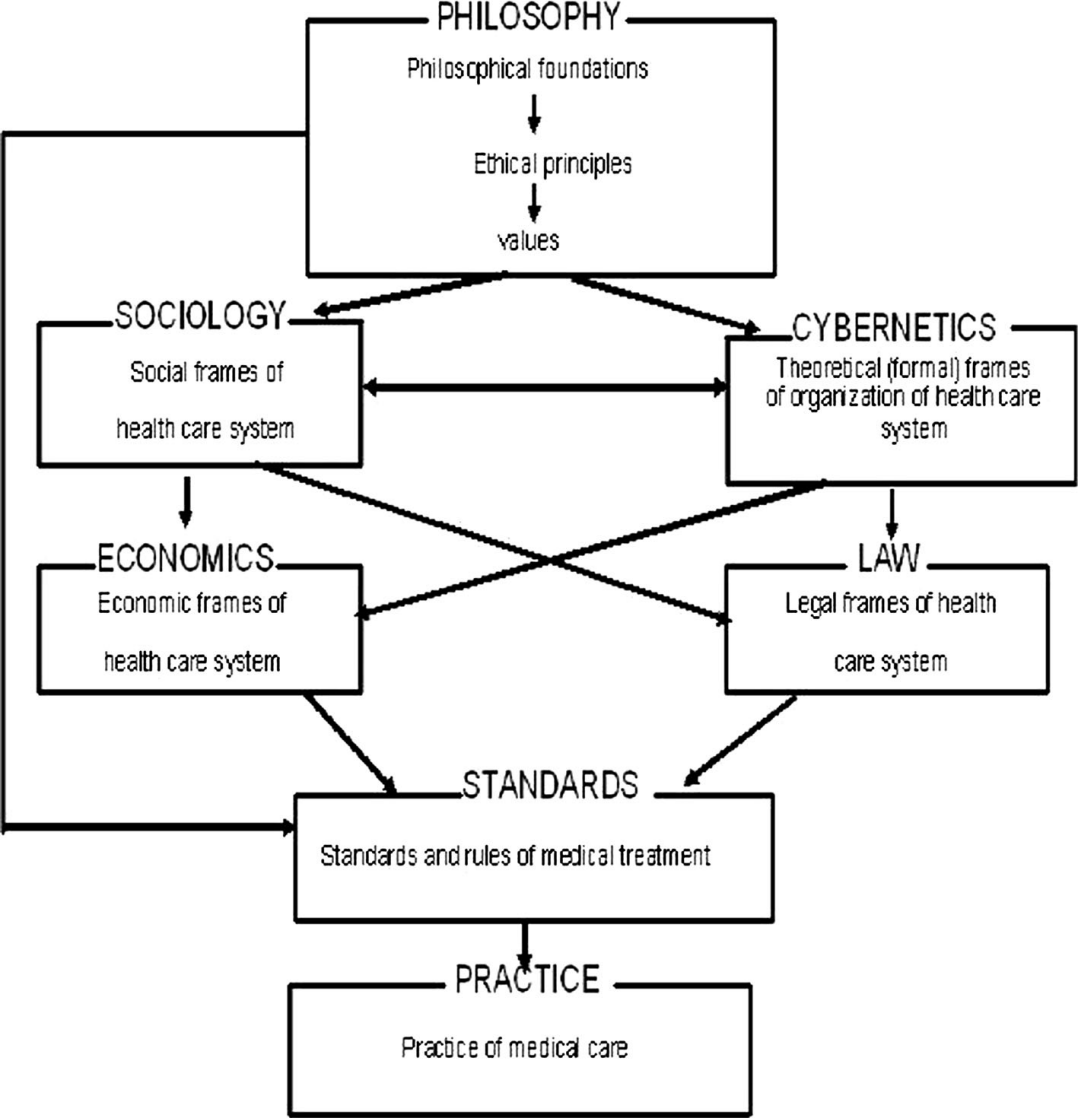
The levels of the analysis and relations between them

The paper is organized in the following way. In the next section the possible levels of studies of health care systems (see Fig. 1) are specified and discussed. Relations between them are also analyzed. The motivations for the conducting studies that concern theoretical, first of all philosophical bases of health care, are discussed in the subsequent section. In the following section the previous attempts to base health care system on the values that are derived from philosophy are presented. Then, the personalism as the possible basis of health care is presented, as well as theoretical frames of the system based on values derived directly from personalistic philosophy. The basic types of the existing health care systems are analyzed within the frames of the introduced proposal.

## The levels of studies of health care systems

The studies that concern health care can be conducted on the following levels:

- philosophical level,
- cybernetic level in which the studied object is regarded as a cybernetic systems, i.e. the system situated in its environment, communicating with it and processing information in a specific way,
- social level,
- legal level,
- economic level,
- the level of standards and rules in medical treatment,
- the level of medical care practice.

The specified levels of studies and the relations between these levels are presented schematically in Fig. 1. The philosophical level is the most basic one. First of all, the commonly accepted general philosophical foundations should be specified. As it has been aforementioned, this choice should be justified thoroughly. Until recently, the constant quest for the truth and common good, worked out in the ancient Greek philosophy and then adopted and enriched by Christianity, were crucial elements of this basis in the European culture. Ethic foundations should be derived from the accepted philosophical basis and then the basic values should be derived from the developed ethics. Beauty, good and truth were the basic values that became foundations of the European culture. The derived values have become the basis of social frames of health care systems i.e. the institutions through which the values are realized in a society. The medieval hospital orders can be put as examples of realization, at the social level, the Christian commandment of charity that is one of the values derived from the Christian ethics. On the one hand, the health care systems should function efficiently and be robust. On the other hand, however, national health care systems are extremely complex. Therefore, they should be implemented and, if necessary, modified on the basis of cybernetic analysis. The cybernetic (systemic) approach to health care systems has not been common so far and only very few researchers are aware of the necessity of such studies. Thus, the papers in which this topic is considered have pioneering character (Bar-Yam 2006; Bielecki and Nieszporska 2016; Bielecki and Stocki 2010; Fahey et al. 2004; Mlakar and Mulej 2007; Trochim et al. 2006). The problems concerning flows of information between modules of the system, decision making, risk and knowledge management are the standard topics in social and economic cybernetics (Bar-Yam 2006; Engemann and Miller 2015; Lin et al. 2014; Sanchez et al. 2015; Val et al. 2014; Yager 2015) and can been easily adapted to health care. The theoretical rules, obtained from the cybernetic analysis, that satisfy the accepted ethic values determine the ways the health care can be realized in a specific society. On the other hand, the specificity of the society determines types of cybernetic systems that can be models for the health care in this society. Both the economic and legal frames of health care systems are determined by the type of a cybernetic system which has been chosen as the systemic basis of the health care system. Specificities of its implementation in the society are also determined by the type of the cybernetic system. Standards and rules of medical treatment are based directly on economic and legal realities, as well as on ethical values, and they determine explicitly everyday health care practice. The holistic studies have to consider all of them, as well as the relations between them. These relations are constituted by the fact that the principles, values and standards obtained or assumed at higher levels determine, not necessarily unambiguously, the principles, values and standards on lower levels. The levels sometimes can affect each other, mutually, in the aforementioned sense.

First of all, in the studies the philosophical system, which will become the starting point for the considerations, should be assumed. This choice should be justified thoroughly. Cybernetic and social levels, that affect each other, are subordinated directly to the philosophical level in the aforementioned sense. Both the economic and legal levels are subordinated directly to both the cybernetic and social levels. The level of standards and rules in medical treatment is subordinated directly to the economic and legal levels. Furthermore, the values derived from the ethical principles on the philosophical level affect the level of standards as well. The level of standards is superior to the level of practice in medical care.

## Motivations

The concepts of human dignity and subjectivity were developed in the European culture on the basis of Greek philosophy, Roman law and Christian ethics. These concepts have been worked out for ages and affected various aspects of people’s life more and more. Contemporary, they manifest themselves as commonly accepted human rights, specified as constitutional regulations in many countries and as the rules specified in documents of the European Union and the United Nations. They have numerous practical implications that concern, among others, the aspects connected with medical care and health care systems.

Diseases, first of all mental, prostrating and terminal ones, are not only the sources of extreme suffering but also, usually, they put patient’s dignity and subjectivity in jeopardy. Therefore, it is not sufficient to work out effective medical treatment but it is necessary to base the health care system on such rules that protect the aforementioned values. The problem is connected with an access to medical help, the relation between a hospital staff and a patient and a social status of the bedridden. Dignity has its origins in religious thinking and human rights and has been developed as a key concept in such clinical fields like palliative and long-term care (Delmar 2013). Therefore, the protection of patient’s dignity is the fundamental value of caring in professional nursing (Jacobs 2000; Rundqvist et al. 2010). According to the definition by Kant, dignity is also associated with being able to cope alone and being independent of others’ help (Jacobson 2009). The independence of others’ help and self-dependence, combined with self-determination and the opportunity to choose and take responsibility for one’s own life, represent a value and philosophy of life that, taken together, may be characterized as self-management (Bauman 2000; Stabell and Naden 2006). The meaning of the above-mentioned, self-managing values, is that the patient is respected as the master of his own life from the patient’s perspective. Thus, dependency, the feeling of being a burden to others, and being neglected as a person with feelings, expectations and values point to a violation of dignity (Delmar 2013). It is necessary to derive these rules from a proper theoretical foundations in order to create a robust health care system.

The concept of human dignity and subjectivity has influence on some practical applications. The respect for human dignity, like many other fundamental moral attitudes, is acquired by most people as a part of living in a contemporary community (Badcott and Leget 2013). This respect causes that certain standards of systems solutions are demanded to be apparent and imbued that implies a lot of problems the health care systems are faced. The foundations on which health care systems should be based on and their implications are discussed in numerous publications. Let us recall a few of them.

Patients today have little choice about ways of treatment and they have no possibility to make informed decisions given the limited information available to them (Porter and Teisberg 2004). Patients could make the best decisions if they were accurately informed about their choices and risks. This is why information campaigns and education of patients, including, for example, medical literacy training, should be important elements of the system. This may happen only if patients are given back the responsibility to make decisions about their care and a significant portion of costs associated with those decisions (Spicer 1994). Medical ethics can no longer rely on an abstract and formalistic conception of the moral agent, i.e. of the predictable, judicially impeccable and rationally immaculate sort of person, as is the situation in many contemporary medical ethics text-books. On the contrary, medical ethics nowadays requires a typological framework capable of giving an account of all existing particulars of the object in question. In other words, what is needed it is a typology of the whole i.e. the typology which would be able to account for all possible varieties of ‘patientness’—of disease induced suffering—with which medicine is morally obliged to deal. The subjective standard would require the physician to inform the particular patient about all important aspects of his disease. Proponents of the subjective standard argue that the patient’s right to make decisions is not adequately protected by any other standard. If patients have a right to make idiosyncratic choices, they may need information that would not be considered significant by reference to the standard of a reasonable person or the standard of a professional consensus. (Solbakk 2014). This standard focuses on the information the ‘reasonable person’ needs to learn about risks, alternatives, and consequences. The legal test under this standard for determining the extent of disclosure is the significance of information to the decision making process of the patient. The patient, rather than the physician, is the judge of whether the information is important. Thus, the right to decide what information is pertinent is shifted away from the physician to the patient (Faden and Beauchamp 1986).

Health care system dysfunction, mentioned in the previous section, cannot be overcome without interdisciplinary studies that allow us, among others, to work out the theoretical foundations on which the health care systems should be based. On the one hand, such general branches as philosophy and cybernetics should be applied—see Fig. 1. On the other hand, the solutions found for other social problems should be adapted to health care systems. It seems that, at least, the studies concerning common exploitation of natural resources (Dietz et al. 2003; Janssen et al. 2007; Ostrom 2009) and both the studies and experiences of the Polish Solidarity movement, that promoted the equality and dignity of all, the centrality of participation, opportunities for the poor, and the insistence on life in truth (Beyer 2007). All of these can be a good starting point for studying not only the analogous problems in health care systems but also philosophical foundations of the systems, the more the ethical aspects of Solidarity phenomenon were studied as well (Tischner 1992). The previous attempts to base the health care system on robust theoretical foundations are far from the satisfactory ones. They are summarized briefly in the next section.

## The state of the arts

Since the humankind started forming such social formations as tribes, cites and, in the end, countries, the single persons or specific structures emerged in order to treat people and prevent diseases. In the ancient world temples were the only places of gathering knowledge and therefore they became the only centers of medicine which had had simply practical character and were not implied by ethical, religious or philosophical foundations (Austin 2014; Sauneron 1960). In the ancient Greece, however, medical knowledge was connected with philosophical systems worked out then in Greece (Hammond 1986) and, therefore, it referred not only to ethics and religion but also to politics as well as in the ancient Rome where, for instance, soldiers were the group that was under special health care because of their role in the ancient imperium (Risse 1999; Szumowski 1949, 2008). In the cultures of great religions such as Buddhism, Christianity, Islam, health care was based on ethical foundations which caused the fact that medicine and health care were practiced widely in monasteries and temples (Ratanakul 2004; Syed 2002; Taheri 2008).

In the Middle Ages the ancient medical knowledge was stored in Christian monasteries where the poor, incurably and terminally ill people were looked after. Since the sixth century, according to the saint Benedict principle: *infirmorum cura ante et super omnia adhibenda est* (*the care of the sick should be put forward everything*), monks have taken care of ill people in *hospitium*, *hospital* or *infirmarium*. Such institutions were supported, legally and financially, by the rulers. In such a way the state-run institutions and government organs were gradually taking over the role of the protectors that took care of citizens. In such a way foundations for the public health care system were laid. On the one hand, activities of the state-run institutions, such as organizing of a system of sanitary control to combat contagious diseases, by using observation stations, isolation hospitals, and disinfection procedures, were caused by ethical rules implied by the religion of the country. On the other hand, the state was interested in keeping farmers, craftsmen, soldiers and all useful citizens in good health.

It was in Germany, at the end of the nineteenth century, more specifically in 1883, when the foundations for the welfare state were laid. It was clear to Bismarck and his contemporaries that the only way to protect individuals from catastrophic health problems was shouldering the risk for the whole community (Sawicki and Bastian 2008). It was the beginning of the social welfare infrastructures, including health care, in the all the European countries. Nowadays these types of systems are challenged by crucial economical problems.

In the twentieth century not only expansion of scientific studies and technological applications in medicine but also a new approach to the organization of the health care system as well as to main ideas in medicine can be observed. According to the previous theories *the aim of medicine is surely not to make men virtuous; it is to safeguard and rescue them from the consequences of their vices* whereas currently it is assumed that *the present wave of idealistic health promotion*, (…), *must be exposed* *to thorough scientific*, *moral and philosophical scepticism* (Kelly and Charlton 1992). In such a holistic approach complex character of health, which is one of the basic human rights, is assumed. Moreover, it can be said that *health has been considered a socio*-*psychosomatic phenomenon* (Meyer-Abich 2005), and in the contemporary societies it is not an individual matter but it remains in causality in both animate and inanimate environment. However, regardless of the approach to health as such, *the enjoyment of the highest attainable standard of health is one of the fundamental rights of every human being without distinction of race*, *religion*, *political belief*, *economic or social condition* [*The Right to Health*, Fact Sheet No. 31, World Health Organization, Office of the United Nations High Commissioner for Human Rights, p.1].

Contemporary, in western culture, freedom of choice, including decisions concerning health, respect to human laws and equality are unarguably accepted, at least declaratively, as absolute rules that are the basis of societies. Despite that, however, even in health care, which is a sector that should refer strongly to these values, the aforementioned values are, in practice, neglected and sometimes, contradicted—for instance, in the context of looking for new solutions. Nevertheless, it seems that in recent years the awareness of necessity of starting discussion about a philosophical basis of health care systems increases (Hofmann 2012; Oduncu 2012; Simonstein 2012).

In the paper (Oduncu 2012), however, apart from a very vague declaration that the German health care system is based on the fundamental principle of solidarity and it provides an ethical and legal framework for implementing equity, comprehensiveness and setting the principles and rules for financing and providing health care services and benefits, financial and structural analysis of the abovementioned system dominates. The basic feature of this system is to supply medical care in a strongly regulated environment in which there are two main goals: the first—financial balance and the second one—maintaining social solidarity in its ethical dimension (Oduncu 2012). The only conclusion is that those who are fully employed help to pay for those who are not yet or any longer employed. Younger and healthier individuals cover the costs of a part of the services that are received by those who are older and less healthy. Those who are single and childless pay for some of the services that are received by those with families and children and, finally, males help to pay for some of the services that are received by females because of their women’s higher gender-specific risks (Henke et al. 1994; Oduncu 2012).

In the Israeli justice, equality and responsibility of the state for the health of its citizens are system the values that are at the very roots of its institution. This implies that *the overriding goal of the health system is the assurance of the right to health services of all individuals in a just and equitable manner* (Simonstein 2012). This statement also reflects the patient’s right that was introduced in 1996 in Israel and has contributed to the value of the right of all people to get reasonable health care. In addition, the nature and the achievements of the health care system in Israel has deep roots in its foundation as a general consensus that society, as a whole, is responsible for health of its citizens (Rosen and Merkur 2009; Simonstein 2012).

All three Scandinavian countries developed the principles of health care systems by a government commissions with members of their parliament and professionals (Hofmann 2012). In Sweden, the Parliamentary Priorities Commission outlined in 1992 three basic principles for priority setting: human dignity, needs and solidarity, and cost effectiveness. It turned out, however, that very few of the medical and administrative staff in the local health care service had heard about these ethical principles and regulations. What is more, several local attempts have been made to limit health services in XXI century. In 1996 the Danish Council of Ethics presented basic values for the health service: equality, solidarity, security and safety, and autonomy. It turned out that the goals implied by this basis may be incongruent but no methods for handling that was provided (Hofmann 2012; Holm 2000). The Norway health care system is based on ideals of equal access and solidarity with the vulnerable. Since 1985 a few committees have been established and they gave the government a series of documents, many of which have not been followed (Hofmann 2012).

To sum up, it seems that in Europe the robust philosophical basis of health care systems was systematically founded only in the Middle Ages and then it was derived directly from the Christian ethics. Since the end of the nineteenth century the idea of the welfare state began to spread in Europe, first in Germany. This led to a welfare state mentality which is common in the contemporary Europe. The discussion about values as a basis of health care systems was started in some European countries at the end of the twentieth century and some values have been declared to be the basis of the health care systems. On the one hand, they are chosen arbitrarily without any profound philosophical reflections. On the other hand, there are many difficulties to implement them as integrative parts of the systems and, therefore, they often remain only as declarations. In the theoretical papers (Hofmann 2012; Oduncu 2012; Simonstein 2012) values are considered as the starting point for studies. Nevertheless, it leads, first of all, to economic analysis of the health care systems.

## The proposed philosophical basis

As it has been mentioned in the first section, the choice of the philosophical basis of the health care system should be justified carefully. The twentieth-century personalism is a stream in philosophy in which human person is superior to historical, political and socio-economic aspects. It is the main basis which is put forward in this paper as the foundation of the health care system. This stream in philosophical thought put emphasis on the dignity of a human as a person, his subjectivity and on the properties in a human that are irreducible to material objects and natural instincts (Crosby 2004; Maritain 1947; Seifert 1989; Wojtyła 1978). Thus, the personalism is such a philosophical system in which human dignity, accepted commonly in the contemporary western culture, has been worked out most comprehensively.

The twentieth century personalists, which Maritain, Mounier, Nedoncelle and Crosby are regarded as the leading representatives, rooted their studies deeply in Christianity, referring strongly to theological aspects. Theses that concern to subjectivity and dignity of the human person were derived by them from the fact that the human being is created by God in His own image or from other strictly theological aspects, for instance how God relates to us (Crosby 2001, 2004, Chapter 1; Maritain 1964, pp. 101–110). This was the main topic of their studies which were referred by them directly to ethics of the individual. Practical implications, however, were not worked out apart from very general considerations. Nedoncelle, for instance, proposed a thesis, that each form of social organization should not reject the person’s rights neither refused the person his or her value (Nedoncelle 1957). Mounier, similarly, stated that the person is free and creative and, as such, should be available for others in communication and community. This should be realized at the state level—the state is for the people, not the people for the state (Mounier 1957; Copleston 1974). Maritain was the one among the aforementioned philosophers who refers to the social aspect of personalism most frequently but rather generally, similarly to the others. He stressed how striving for community is crucial for a person. This striving to include in the network of social contacts is connected with the need for love and exchange of cognition. Both these aspects are strictly connected with providing the person the possibility of existence and complete self-realization. There exists basic rights of a human that should be respected by community (Maritain 1940, pp. 56–88). The last statement harmonizes with the postulates specified by Nedoncelle.

Wojtyła, who was also one of the leading personalist of the second half of the twentieth century, was in a specific situation. In spite of the fact that he was not only a catholic priest, but also a bishop and the pope, he was the philosopher among personalists, who was capable to leave aside theological aspects, first of all in *Acting Person* (Wojtyła 1969), basing mainly on phenomenology and refers strongly to the problem of self-experience by a human. In those his encyclical letters, even, that strongly concerned sociological aspects (John Paul II 1981, 1991, 1995) the theses were rooted not only in theology, but in universal aspects of personalism as well. As a result he strongly referred to ethics in its universal aspect. Furthermore, he was the only personalist, who not only analyzed theoretical aspects of human dignity but also possibilities of applications for the personalistic ideas to these domains of existence that are connected with social relations. This topic was considered directly in his philosophical works (Wojtyła 1969, 1976a) as well as in the encyclical letters *Laborem Exercens* and *Centesimus Annus*. As a philosopher Wojtyła stressed the role of philosophic ideas in shaping various attitudes of various social environments including medical circles to which he frequently turned directly. In his texts directed to the medical environment he stressed the need for love, a sense of togetherness, solidarity and a respect to natural human dignity. He reminded how important are ethical aspects of medical profession in which *deepest inspiration and strongest support lie in the intrinsic and undeniable ethical dimension of the health*-*care profession*, *something already recognized by the ancient and still relevant Hippocratic Oath*, *which requires every doctor to commit himself to absolute respect for human life and its sacredness* (John Paul II 1995, paragraph 89).

Guidelines for health care formulated by Wojtyła, in particular superiority of human subjectivity and dignity, remains in keeping with official documents of the World Medical Association, in which highest respect for human life and primacy of good of the human over interests of science and society were declared directly (World Medical Association Declaration of Geneva 1948; World Medical Association Declaration of Helsinki 1964). This is a strong argument for the thesis that the Wojtyła’s approach to personalism, in particular social applications aspects, is not only rooted in Christianity but has strong universal aspect as well. Thus, it could be a good basis for creation of ethical foundations of social organizations and public institutions in contemporary states in the European cultural circle in which religion neutrality as well as neutrality in terms of outlook is strongly declared.

The sense of identity is a very basic one for human individuals. It is inseparably linked with the consciousness of choices and perseverance in pursuing goals. The psychological point of view defines it as the sense of subjectivity (Henriques et al. 2005). Such an attitude results in high self-esteem which, on turn, refers strongly to human dignity. The aforementioned properties contribute to human moral values, rights and obligations, honor, and to proper relations with the society (Shultziner 2007).

The subjectivity and dignity of a human are accepted commonly in the contemporary western culture. This specific concept of a human according to the personalistic approach has implications concerning the role of an individual in the society (Wojtyła 1977). The common relations between the members of the society, and mainly the activity and cooperation among the members of the society, should be rooted in the aforementioned human subjectivity. The specificity of the relation between an individual and the society in the context of human subjectivity implies not only specific social norms, rules, and obligations but also the principles of action (Coleman 1988).

Man is a rational being. On the other hand he is, by his nature, a social individual (Wojtyła 1969). It means, that in the very essence of him, he is determined by acting and he gets fulfilled in cooperation with the others. Such cooperation is a participation in common acting which allows an individual self-realization. It is connected with references to ethical values and self-determination. Thus, it can be concluded that willing is nothing but a spontaneous turning toward a value which finally transforms a human sheer desire into a decision (Wojtyła 1979). Human acting is connected with the necessity of making choice and decision. They should be in harmony with a human will which is a manifestation of human freedom and subjectivity (Dietz et al. 2003). Human’s will is rooted in values that are both the basis and aims. The quest for these aims, as well as their defining, make the way for self-manifestation and self-creation which is legitimate if is realized within the absolute system of values.

Thus, strong relations between subjectivity (free will, among others) and participation is strongly stressed in personalistic philosophy. They are determined reciprocally and they both imply freedom of an individual (Wojtyła 1977). This freedom is considered as the possibility to be a prime mover in an individual initiative. Thus, activity is the essence of personalism and implies participation as not only natural right but also an obligation of each individual. The main idea of participation can be expressed in the following way: *They are not only* “*other*” *in their relation to the* “*I*”, *but each one of them is at the same time a* “*different I*” (Wojtyła 1977). The fact that everyone is a subject and a free human being, equipped with social aspect of his life and, as a consequence, acting in societies and communities, is human’s natural right. That fact should be the basis of social systems, first of all the health care ones.

The premise that the person, as it is understood in personalism, should be the reference point in all the considerations that concern social and political problems, is the main assumption in this paper. On the one hand, personalism puts stress on the human subjectivity and dignity. On the other hand, it emphasizes the social aspect of human individuals. The personalistic concept of the person is rooted deeply in the experience and philosophy of the West. Not only the western philosophers obtained very similar conclusions when they considered the problems concerning a human individual as such but also psychologists and sociologists (Fromm 1976; Maslow 1962; Rogers 1961).

To sum up, it seems that personalism is the most universal theory that concerns the person among the existing philosophical systems of the West. This means that on the one hand, the concept of a person as such has been worked out in details within its frames. On the other hand, many other currents in the Western philosophy, both connected with Christianity and irreligious ones, give very similar answers concerning the nature of a human being and the implications regarding its social role. The crucial question is what are the practical consequences of personalism for the social activity and organization of life in the social aspect. This is a particular case of a classical philosophical problem concerning the relation between theory and practice (Wojtyła 1978). Let us follow the proposed schema—see Fig. 1, and firstly, let us specify the philosophical foundations derived from personalism that will be used as the basis for ethical rules applied as the principles of the health care system organization. A human being is the person—it is the most crucial statement in personalism. Each person has their unique value which is manifested, among others, as the person’s subjectivity and dignity which are the immanent properties of the person as well as free will. The above general foundations, i.e. subjectivity, dignity and uniqueness of the human person, are the sources of the following ethical principles. As a subject the human has the inalienable right to freedom (Crosby 1984; Styczeń 1985) which is, among others, a source of human causative potential (Wojtyła 1969, chapter 2). This potential is realized in participation in common (social) enterprise, in particular in participation of the person in each decision and enterprise that concern him or her (Wojtyła 1969, chapter 4). The human not only realizes himself and his auto-teleology by participation in community, first of all in cooperation with other human beings, but also enriches the community by his participation (Wojtyła 1969, 1976b). This allows to effectively solve the common problems by activation of the individual initiative (Hayek 1949, 1960). Thus, the participation principle is rooted in freedom which is, in turn, implied by human subjectivity. The human dignity implies that human person is superior to, among others, economic aspects. This implies, that a person cannot be treated instrumentally which means, among others, inalienable right to life and medical care. To sum up, respect of personal dignity, subjectivity and, as a consequence, the right to life as well as guarantee for freedom for each person are the most imported values derived from personalistic assumptions. These assumptions as well as the fact that the human exists in community imply also solidarity as the basis of community. The common good generates participation whereas solidarity serves the common good (Wojtyła 1969). In health care solidarity has additional aspect—solidarity with the needful people.

The specified values imply the principles according to which the health care system should be organized.

1. Everyone should have an access to the health care.
2. Everyone should have as large possibilities of choices in medical treatment as possible. This implies that everyone should have a broad access to information and knowledge that are crucial in the individual decision making process concerning the choice of medical treatment. Furthermore, the freedom of wide spreading information, among others via the Internet, as well as broad discussion on the possible methods of treatment, and education both at school and in the communities, should be guaranteed.
3. Health care system should enable both health care staff and patients active participation. That means the health care system should allow both the medical staff and the patients to implement the created innovations.
4. The access to all the information, that concern both the performance the whole system and the way its subunits act, should be ensured. Each participant should have an access to this information as well as possibility to create new pieces of information and place them into the public information space. This is connected with the point (2).
5. Both the principles of the system performance and the ones that describe the way it can be modified should be specified explicitly and clearly.

The main role of the above listed rules is to provide an individual with the possibility to realize their subjectivity and freedom in the health care system by ensuring maximal possibilities being active within the system, according to their specificity, among others their role in the system, and expertise. Such an approach is consistent with the concept of participation as the way of realization of the aforementioned subjectivity and freedom (Wojtyła 1969, 1977). It can be manifested, for instance, in the creation of the codes of ethics. Nowadays, only professional associations and occupational groups create the codes of ethics with the purpose of guiding their members, protecting the service users, and safeguarding the reputation of the profession (Spielthenner 2015). According to the principle of participation, patients should be included into the debate on ethical values in medicine.

The abovementioned way of participation strongly refers to solidarity. *Man is always in solidarity with somebody and for somebody* (Tischner 1992). Conscience, which is a natural “ethical sense” of man is the main source of solidarity. Solidarity is one of a number variants of the interpersonal relations. The cooperation of a group of people is not easy and, as it is implied by the results of long-term studies, there is a few factors that affect the final success of a collective acting on the social level (Ostrom 2010). There are, among others:

1. The number of participants (as the group size increases, the cooperation level decreases).
2. Shared property (subtractive) vs. public goods (more cooperation occurs when a public good is in question, and less cooperation with the shared property goods).
3. The heterogeneity of participants (generally speaking, more diverse groups are less likely to cooperate).
4. Face-to-face communication (increased face-to-face communication correlates with more cooperative behaviour).
5. Information about past actions (more information about past actions is likely to lead to more cooperation).
6. The type of links between people—direct links tend to lead to more intense cooperation.
7. Entry and exit—the situations where parties can easily withdraw may lead to more cooperation.

Let us comment the specified rules in the light of two concepts of liberty: liberalistic (Gray 1993, 1998; Hayek 1949, 1960) and personalistic (Crosby 1984; Styczeń 1985; Wojtyła 1969). According to the liberalistic concept the freedom of a person should be limited only by the freedom of the other person. The problem is on which level this rule is taken as the basis. If this principle is taken only at on national level as the basis for the law, then it should be complemented on the social and individual levels, first of all in their ethical aspects. In such a case the concept of freedom of an individual, including the social relations, can be specified more precisely by personalism. Within the personalisic frames, the individual freedom is not the acceptance of doing as someone like, as in some concepts of liberalism, but intentional giving oneself for disposal of the good, in particular the common good (Crosby 1984; Styczeń 1985). In such a perspective freedom means, first of all, the possibility of doing the deeds that should be done from a moral point of view. It is simply the activation of an individual initiative. Study of the person in the context of liberty as a source of causality is the basis of understanding a human being as the active subject (Wojtyła 1969, chapter 2). Activity in a community, in particular the joint activity, has a specific aspect of self-realization (autoteleology) because a human, in the very nature of things, is a social being (Tatarkiewicz 1930; Wojtyła 1979, chapter VII).

The transition from the abstract norms to practice is neither simple nor automatic. It is not sufficient to know the norm—it is also necessary to approve it (Buttiglione 1991). In the case of the health care a norm has to be accepted at least on three levels: the national, social and individual ones. On the national level the legal frames that have the approved norms as the basis have to be created. The health care system should be organized within these legal frames, which is a derivative of the theoretic basis. The approval on the individual level is necessary because individuals should take the active part in creation, sustaining and development of the created system. On the social level the approval is necessary for, at least, two reasons. First of all, the aforementioned system creation and sustaining can be realized only by joint, i.e. social, activities. Furthermore, the community that realizes the shared tasks has broad possibilities to detect, prevent and eliminate some destructive activities of the dishonest participants. It turns out that in the groups which learn from experience and adopt a norm of conditional cooperation, humans can cooperate to produce shared, long-term benefits. This topic has been worked out in the context of the common use of natural resources (Vollan and Ostrom 2010). Common acting allows people to minimize risk, to increase potentiality and accumulation of reserves which can be analysed precisely within the frame of the systems theory (Bielecki and Stocki 2010; Bielecki and Nieszporska 2016). The aforementioned decreasing of risk is connected, among others, with the resistance of the common (collective) systems to disruptions which implies that the participants in the system trust it. These problems are well worked out in the context of the joint exploitation of natural resources (Janssen et al. 2007; Vollan and Ostrom 2010) and the results can be adapted, probably, to the health care systems. Furthermore, the Polish experiences of joint acting based on solidarity in the context of social movement (Beyer 2007; Cirtautas 1997; Meardi 2005; Osa 1997; Tischner 1992) can also be tried to be adapted.

To sum up, in this section the philosophical foundations, ethical principles, basic values and the principles according to which the health care system should be organized are proposed. The first three belong to the philosophical level of the analysis of the problem of the foundations on which the national health care system should be based whereas the fourth one is the basis for theoretical frames of the health care system organization—see Fig. 1.

## The existing systems analysis within the frame of the proposed basis

In general, in the existing health care systems four main types can be distinguished (Bielecki and Stocki 2010; Bielecki and Nieszporska 2016). Let us analyze them briefly in the frame of the philosophical basis, proposed in this paper.

The first one is the health care system fully paid by the patients. In this type of the health care system, called by economists residual, liberal or private (Beresniak and Duru 2008), a patient pays directly for every medical service and receives it immediately. Medical care is treated as an ordinary good, which may be purchased or not, depending on the needs and the financial means at the patient’s disposal. This kind of a system covered most of the national health care in most European countries in the nineteenth century. Nowadays, the systems of the veterinary service and, in most cases, the dental service are organized according to this model. In this type of a system the individual’s freedom is respected but its dignity is violated. The fact that medical care is treated as the good implies that the human health is treated in the same way. This means, among others, that the subsystems of the health care system are not interested in the patient’s health but in his being treated.

The second one is the system in which the health service costs are covered by insurance companies in which a patient could insure himself or not according to his will. In such a system patients buy packets of health care services from insurance companies that cover the treatment costs within the services covered by their insurance policy. The patient freedom is respected and, theoretically, so is their dignity. In practice, however, the insurance companies often try to find any excuse to refuse to cover the costs of the treatment. Oftentimes the conditions of insurance are formulated in an unclear way with the purpose to make their interpretation difficult. Thus, the patient has to carry out a specific game with the insurance company which often ends in the court. It is clear that such events are an affront to common sense of dignity and to the right to health care.

Health care systems in the welfare states are based on national insurances. As it has been aforementioned, the first system of this type was founded by Bismarck in the nineteenth century in Germany. Not only national health insurance of workers and their families in the case of sickness or disablement but also retirement pensions were included into them. The overriding aim of the introducing of the system was different: *the rationale underlying the Bismarckian policy was the prevention of the socialist challenge to the authority of the state by the industrial proletariat* (Porter 1999). Bismarck himself called his social politics a practical Christianity but he took care neither of the women nor of the children that worked in factories. Workers were not treated as a political or social subject by Bismarck (Mommsen 1959). The health policy in Germany before the First World War is the example of political solution that was effectively incapacitate or even enslave citizens that was already criticised (Belloc 1912). Although the citizens were under the health care, they were treated instrumentally by the government for whom the strengthening of the national institutions was the only aim (Mommsen 1959). Nowadays, a welfare state is responsible not only for the legal aspect but also for the organizational and financial aspects of the health care (Lameire et al. 1999). Such a system is typical for the Western Europe. It is opposite to the foundations of democracy in which decentralization is one of the fundamental principles. It is because decentralization is *synonymous with redistribution of power*, *resources*, *and administrative capacities through different territorial units of a government and across local groups* (Agrawal and Ostrom 2001). The role of the contemporary welfare states is dysfunctional not only from the economic point of view. The philosophical and ethical studies in most cases also lead to the negative appraisal of the idea of the welfare state. The social teaching of the Catholic Church can be put as the example:

…excesses and abuses, especially in recent years, have provoked very harsh criticisms of the Welfare State, dubbed the “Social Assistance State”. Malfunctions and defects in the Social Assistance State are the result of an inadequate understanding of the tasks proper to the State. Here again the principle of subsidiarity must be respected: a community of a higher order should not interfere in the internal life of a community of a lower order, depriving the latter of its functions, but rather should support it in case of need and help to coordinate its activity with the activities of the rest of society, always with a view to the common good. By intervening directly and depriving society of its responsibility, the Social Assistance State leads to a loss of human energies and an inordinate increase of public agencies, which are dominated more by bureaucratic ways of thinking than by concern for serving their clients, and which are accompanied by an enormous increase in spending (John Paul 1991; see also Rhonheimer 2003).

Let us notice that in the above passage the principle of subsidiarity is stressed strongly and it corresponds to the approach presented is Ostrom’s papers (Agrawal and Ostrom 2001; Ostrom 2009, 2010). To sum up, the centralized systems in the Western European welfare states are far from being perfect regarding their functional aspect. Furthermore, as it has been aforementioned, they have some ethical shortages. It should be admitted, however, that they enable all the society to being provided with free medical care.

A fully centralized health care systems were typical of communist countries. They are still functioning in some of the post-communist countries, including Poland. It leads to total enslaving patients. The system is planned and controlled centrally. The regulations for refunding the costs of medicaments are set arbitrarily, which naturally makes pharmaceutical companies lobby for their products. The citizens are forced to pay taxes (so-called national health insurance) for the national health care, which does not provide them with sufficient medical care and, in many cases, in particular those ones that require specialist interventions, the patients have to cover some costs (Bielecki and Stocki 2010; Bielecki and Nieszporska 2016). In general, the centralized financing deprives the patient of the responsibility for his or her treatment. It is attributed to the doctor or the hospital. The patient is a passive receiver of the service.

To sum up, all the existing health care systems are more or less dysfunctional (Bielecki and Nieszporska 2016). Furthermore, they either pass over the problem of philosophical foundations or consider it only declaratively. Even in the theoretical studies (Badcott and Leget 2013; Delmar 2013; Jacobs 2000; Solbakk 2014; Spielthenner 2015; Stabell and Naden 2006) the philosophical basis is chosen arbitrarily without profound justification.

## Concluding remarks

The philosophical foundation of health care system is proposed in this paper. The postulated foundation is based on personalism according to which human dignity, subjectivity and free will is stressed significantly. Furthermore, personalism implies directly the person’s right to participate in all the events that concern them. Since these values and the principles should be taken into consideration, as the crucial ones in health care system, it is obvious that personalism is the proper base. What is more it is in accordance with the principles concerning the health care derived, both contemporary and in the past, from various religious systems. Furthermore, personalism not only is in resonance with social teaching of the Catholic Church but also it is in resonance with the western philosophers who made a considerable contribution to it. Moreover, personalism uniquely constitutes a universal ethical system founded on human dignity, subjectivity and freedom. Then, from the proposed general foundations, some ethical principles and basic values have been derived. The specified values implied the principles according to which the health care system should be organized. Unfortunately, none of the existing health care systems is based on the proposed philosophical basis. Furthermore, the studies that concern this basis are rather marginal. It seems that only the hypothetical health care system, proposed in Bielecki and Stocki 2010 and analyzed in Bielecki and Nieszporska 2016, can ensure realization the postulates implied by the proposed basis.

The problems of implementation of the postulated principles have been considered in this paper as well. These considerations refers strongly to the results obtained by Elinor Ostrom in the aspect of collective exploitation of natural resources. Thus, the analysis has been led not only on the philosophical level but it has also referred to cybernetic, legal and social levels—see Fig. 1.

## Footnotes

1 As the European circle of culture we understand the common cultural and spiritual heritage derived from philosophy of ancient Greece, the legal basis worked out in ancient Rome, Christian ethics, the Renaissance humanism and political ideas of the Enlightenment.
